# Mechanisms by Which Probiotic Bacteria Attenuate the Risk of Hepatocellular Carcinoma

**DOI:** 10.3390/ijms22052606

**Published:** 2021-03-05

**Authors:** Wasitha P.D. Wass Thilakarathna, H.P. Vasantha Rupasinghe, Neale D. Ridgway

**Affiliations:** 1 Department of Plant, Food, and Environmental Sciences, Faculty of Agriculture, Dalhousie University, Truro, NS B2N 5E3, Canada; wasitha@dal.ca; 2 Department of Pathology, Faculty of Medicine, Dalhousie University, Halifax, NS B3H 1X5, Canada; 3 Department of Pediatrics, Faculty of Medicine, Dalhousie University, Halifax, NS B3K 6R8, Canada; neale.ridgway@dal.ca; 4 Department of Biochemistry and Molecular Biology, Faculty of Medicine, Dalhousie University, Halifax, NS B3H 4R2, Canada

**Keywords:** carcinogenesis, microbiome, liver cancer, probiotic bacteria, post-biotics

## Abstract

Hepatocellular carcinoma (HCC) is the most common primary liver cancer and the second leading cause of cancer-related deaths worldwide. Chronic infections with hepatitis B virus (HBV) and hepatitis C virus (HCV), alcoholic liver disease (ALD), and non-alcoholic fatty liver disease (NAFLD)/non-alcoholic steatohepatitis (NASH) are the major extrinsic risk factors of HCC development. Genetic background is pivotal in HCC pathogenesis, and both germline mutations and single nucleotide polymorphism (SNP) are intrinsic risk factors of HCC. These HCC risk factors predispose to hepatic injury and subsequent activation of fibrogenesis that progresses into cirrhosis and HCC. Probiotic bacteria can mitigate HCC risk by modulating host gut microbiota (GM) to promote growth of beneficial microbes and inhibit HCC-associated dysbiosis, thus preventing pathogen-associated molecular patterns (PAMPs)-mediated hepatic inflammation. Probiotics have antiviral activities against HBV and HCV infections, ameliorate obesity and risk of NAFLD/NASH, and their antioxidant, anti-proliferative, anti-angiogenic, and anti-metastatic effects can prevent the HCC pathogenesis. Probiotics also upregulate the expression of tumor suppressor genes and downregulate oncogene expression. Moreover, metabolites generated by probiotics through degradation of dietary phytochemicals may mitigate the risk of HCC development. These multiple anticancer mechanisms illustrate the potential of probiotics as an adjuvant strategy for HCC risk management and treatment.

## 1. Introduction

Primary liver cancer, which has the second highest mortality worldwide [1], is a heterogeneous disease with several types of malignant tumors, including HCC, intrahepatic cholangiocarcinoma (iCAA), mixed hepatocellular cholangiocarcinoma (HCC-CAA), fibrolamellar HCC (FLC) and pediatric neoplasm hepatoblastoma [2]. HCC accounts for 75–85% of all primary liver cancers. iCAA is the second most common primary liver cancer (10–15%) [1], and the occurrence of HCC-CAA, FLC and hepatoblastoma is less than 1% [2]. HCC primarily originates from hepatocytes whereas iCAA originates from cholangiocytes (bile duct epithelial cells) and mature hepatocytes reprogrammed into a biliary-like phenotype [3]. HCC can be broadly divided as non-proliferative and proliferative, with the latter being associated with poor outcome. Around 30% of the non-proliferative HCC cases have mutations in the catenin beta-1 (CTNNB1) gene that activates the β-catenin signaling pathway. The proliferative variant of HCC is more aggressive and associated with high serum level of α-fetoprotein (AFP), expression of progenitor cell phenotype, tumor protein 53 (TP53) mutations, and activation of transforming growth factor β (TGF-β), hepatocyte growth factor receptor (MET), protein kinase B (AKT), and insulin-like growth factor (IGF) 2 pathways [4]. Despite advancements in the treatment of HCC, including intra-arterial therapy, multi-kinase inhibitors and immune therapy, the overall prognosis remains poor [5].

Since the liver is directly linked with the intestine through the hepatic portal circulation, pathogenesis of HCC is associated with negative alterations to the gut microbiota (GM) [6]. This anatomical connection indicates that probiotic bacteria could restore the gut bacterial complexity and colonization resistance to overcome HCC-associated dysbiosis [7]. Probiotic bacteria can stimulate the growth of beneficial short-chain fatty acid (SCFA)-producing bacteria in the GM [8]. SCFA are known to modulate anti-inflammatory responses and regulate cell differentiation and proliferation [9]. Moreover, probiotic bacteria can protect intestinal epithelial function and prevent bacterial endotoxemia by restricting the translocation of gut bacteria and their metabolic products into the liver [7]. Probiotic cell components and probiotic metabolites promote gut epithelial integrity by upregulating the expression of tight junction proteins. The cell surface proteins of probiotic bacteria can attenuate inflammation of gut epithelial cells and inhibit epithelial cell apoptosis to maintain the gut epithelial integrity. Moreover, the ability of probiotics to increase mucus secretion from goblet cells and release of antimicrobial peptides protects the gut epithelium from pathogenic bacteria [10]. Endotoxemia resulting from an altered GM has been identified as a major risk factor for HCC that promotes pathogenesis through chronic hepatic inflammation [11]. Probiotic bacteria, especially *Lactobacillus* sp. and *Bifidobacterium* sp., have the potential to reduce fatty liver and insulin resistance in obese subjects [12] and therefore reduce the risk of HCC [13]. Additionally, the anti-viral activity of probiotic bacteria against HBV and HCV infections could also reduce the risk of HCC [14].

In this review we discuss the probiotic-mediated anticancer mechanisms exclusive to HCC. Current knowledge on the potential of probiotics to prevent HCC pathogenesis promise a future avenue of alternative treatment and management measures. However, clinical studies directly evaluating probiotics in HCC prevention or treatment are limited. Future studies must be focused on investigating the synergistic effects of different probiotics for HCC risk reduction to formulate probiotic mixtures capable of delivering superior anticancer effects. The potential to utilize synbiotics, a mixture of probiotic bacteria and corresponding prebiotics, to improve the efficacy of probiotics and selectively stimulate the growth of beneficial gut microbes [15] is a strategy of cancer risk reduction that must be further studied. The ability of probiotic bacteria to biotransform prebiotic dietary components including polysaccharides [16] and phytochemicals [17] into metabolites with anticancer properties need to be further investigated to formulate effective synbiotics for HCC prevention and treatment.

## 2. Hepatocellular Carcinoma

**Figure 1:**
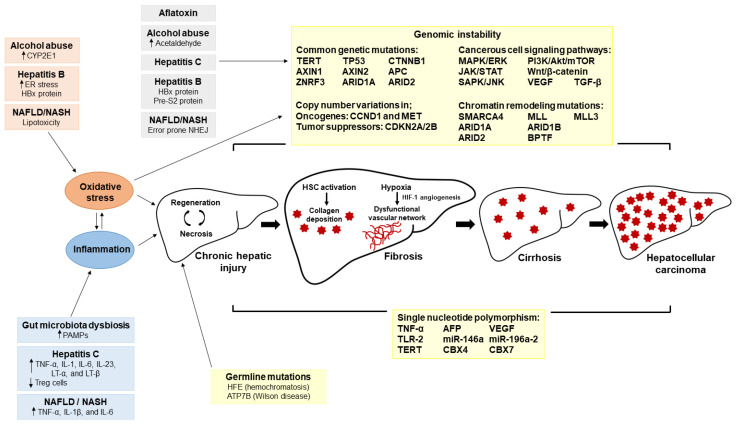
Etiological risk factors and pathogenesis of hepatocellular carcinoma (HCC). Chronic infection with hepatitis B and C viruses, alcohol abuse, non-alcoholic fatty liver disease, gut microbial dysbiosis, aflatoxin, germline genetic mutations and single nucleotide polymorphism are the major risk factors of HCC development. These etiological risk factors can induce chronic hepatic damage through inflammation and oxidative stress. Chronic hepatic injury leads to the activation of hepatic stellate cells and subsequent fibrogenesis. Chronic prevalence of etiological risk factors supports the transition of fibrotic liver into cirrhosis and eventually HCC. Genomic instability introduced by these etiological risk factors contribute to the initiation of HCC and the progression of fibrotic liver into HCC. Abbreviations used: AFP, α-fetoprotein; APC, adenomatous polyposis coil; ARID, AT-rich interaction domain; ATP7B, ATPase copper transporting beta; AXIN, axis inhibition protein; BPTF, bromodomain PHD finger transcription factor; CBX, chromobox; CCND1, cyclin D1 encoding gene; CDKN2A, cyclin-dependent kinase inhibitor 2A; CDKN2B, cyclin-dependent kinase inhibitor 2B; CTNNB1, catenin beta-1 gene; CYP2E1, cytochrome P450 2E1; ER, endoplasmic reticulum; HBx, Hepatitis B virus x protein; HCC, hepatocellular carcinoma; HFE, human homeostatic iron regulatory protein; HIF-1, hypoxia-inducible factor 1; IL, interleukin; JAK/STAT, Janus kinase/signal transducer and activator of transcription; LT-α, lymphotoxin α; LT-β, lymphotoxin β; MAPK/ERK, Ras-Raf-mitogen activated protein kinase/extracellular signal-regulated kinase; MET, hepatocyte growth factor receptor; miR, microRNA; MLL, myeloid/lymphoid leukemia; NAFLD, non-alcoholic fatty liver disease; NASH, non-alcoholic steatohepatitis; NHEJ, non-homologous end joining; NKT, natural killer T cells; PAMPs, pathogen-associated molecular patterns; PI3K/Akt/mTOR, phosphoinositide 3-kinase/Akt/mammalian target of rapamycin; SAPK/JNK, stress-activated protein kinase/c-Jun NH2-terminal kinase; SMARCA4, SWI/SNF related, matrix associated, actin dependent regulator of chromatin, subfamily a, member 4; TERT, telomerase reverse transcriptase; TGF-β, transforming growth factor β; TLR, Toll-like receptor; TNF-α, tumor necrosis factor α; TP53, tumor protein p53; Treg, regulatory T cells; VEGF, vascular endothelial growth factor; Wnt, wingless/int-1; ZNRF3, zinc and ring finger 3.

HCC is a complex and multistep disease with several morphological subtypes [18]. There are multiple etiological risk factors of HCC that dictate the disease progression and pathogenesis (Figure 1) [19]. Many of these risk factors are extrinsic and can be modified by lifestyle changes. Chronic infection by hepatitis type B, C, and D viruses, alcohol abuse, NAFLD and NASH, obesity and diabetes mellitus, liver damage by aflatoxin (mycotoxins) and smoking are the major extrinsic risk factors of HCC development (Figure 1) [19,20]. About 80–90% of patients suffer from cirrhosis before being diagnosed with HCC. The chronic presence of HCC risk factors, especially hepatitis infection, alcohol and NASH, promote liver inflammation that eventually leads to fibrosis and cirrhosis by the activation of liver myofibroblasts [21]. The chronic damage and inflammation in cirrhotic liver causes a high rate of hepatocyte regeneration leading to the accumulation of malignant genetic mutations that initiate carcinogenesis. Only about 20% of HCC cases arise in the absence of cirrhotic conditions [22]. However, the etiological risk factors of non-cirrhotic HCC are similar to the cirrhotic HCC [23].

### 2.1. HCC Etiology

Over 50% of HCC cases worldwide are associated with HBV infection, and individuals with chronic HBV infection are 10–20 times more susceptible to the development of HCC. HBV- induced HCC is driven by the genomic instability created by insertional mutagenesis [24] and the production of the oncogenic proteins Hepatitis B virus x (HBx) and pre-2S/S (Figure 1) [24,25]. HBx promotes cell proliferation, tumor angiogenesis [26,27], oxidative stress-mediated hepatic injury [28] and HCC metastasis by inducing the epithelial-mesenchymal transition (EMT) [29]. The accumulation of oncogenic pre-S2 mutant protein in the endoplasmic reticulum (ER) of hepatocytes causes ER stress and promotes developmet of characteristic ground glass hepatocytes predispose to HCC. The ER stress created by pre-S2 mutant protein can induce oxidative DNA damage in hepatocytes by increasing cellular reactive oxygen species level and upregulating nuclear factor-κB (NF-κB) and cyclooxygenase-2 (COX-2) expressions [25].

Chronic infection with HCV is the main etiological HCC risk factor in most Western countries [30]. HCV infection increases the risk of HCC by 15–20 fold [31], with 75–85% of patients progressing into chronic infection [32]. The core proteins of HCV promote lipogenesis and induce oxidative stress in hepatocytes [33] and significantly influence the cell signaling pathways regulating hepatocyte proliferation and the expression of tumor suppressor genes TP53 [34] and retinoblastoma susceptibility gene (RB_1_) [35]. The nonstructural (NS) proteins of HCV promote liver fibrosis [33] and HCC metastasis [36]. Moreover, HCV increases chronic liver inflammation by inhibiting the production of type I interferon and transformation of CD8^+^ T-cells to T helper type 1 (Th1), Th17 and regulatory T-cells (Treg). Liver inflammation is further boosted by the influx of pro-inflammatory cytokines tumor necrosis factor (TNF)-α, interleukin (IL)-1, IL-6, IL-23, lymphotoxin (LT)-α and LT-β (Figure 1) [37].

NAFLD is positively associated with the HCC pathogenesis and is becoming a major risk factor due to its increasing prevalence worldwide [38]. In NAFLD patients, hepatic influx of fatty acids (FA) induces steatosis [39,40] and lipotoxicity, leading to mitochondrial dysfunction, ER stress and hepatic oxidative stress. Steatosis induces liver inflammation by upregulating NF-κB to produce the pro-inflammatory cytokines TNF-α, IL-1β and IL-6 (Figure 1) [40]. Activation of natural killer T cells (NKT) during NAFLD/NASH promotes steatosis and together with CD8^+^ T cells, induces liver injury as indicted by hepatocyte ballooning and Mallory-Denk body formation. The liver injury caused by NASH can induce damage-associated molecular patterns (DAMPs)-mediated inflammatory responses that activate immune cells (e.g., Kupffer cells) to induce the production of proinflammatory cytokines and localize to sites of damage, thus promoting hepatic inflammation [41]. The transition from fatty liver to HCC is further promoted by oxidative DNA damage, DNA methylation defects (e.g., Salvador family WW domain containing protein 1/Sav1) [42] and reduced expression of tumor suppressor genes (e.g., zinc fingers homeoboxes 2/ZHX2) [43]. In NAFLD and NASH, the DNA damage response (DDR) mechanism is substantially restricted by depletion of ataxia telangiectasia mutated (ATM) through sustained DNA damage [44]. However, the error-prone non-homologous end joining (NHEJ) pathway is intensified during NAFLD-HCC due to increased expression of DNA-dependent protein kinase (DNA-PK) [45,46].

Alcohol abuse and subsequent development of ALD is a major risk factor that accounts for 30% of HCC cases [47]. Pathogenesis of ALD initiates with simple steatosis and progresses through alcoholic hepatitis, fibrosis, and cirrhosis (Figure 1) [48]. Hepatic metabolism of ethanol contributes directly to HCC by promoting DNA adduct formation, oxidative stress, and depletion of retinol and retinoic acid. Increased activity of cytochrome P450 (CYP) 2E1 during alcohol metabolism elevates hepatic oxidative stress and activates pro-carcinogens, including nitrosamines, polycyclic hydrocarbons, and hydrazines (Figure 1). Moreover, increased CYP2E1 activity depletes retinol and retinoic acid from hepatic tissues, disrupting cell growth and trans-differentiation [49]. Acetaldehyde generated from ethanol metabolism acts as a carcinogen by forming DNA adducts (e.g., *N*^2^-ethylidene-2′-deoxygusnosine) [50] and gene mutations [51] (e.g., TP53 tumor suppressor [52]).

Inherited genomic (germline) mutations and SNPs that predispose to chronic liver diseases are intrinsic risk factors of HCC (Figure 1) [53]. SNPs are single base-pair substitutions within coding or non-coding regions of DNA that can alter DNA repair, cell regulation, and immunity and significantly increase the risk of cancer pathogenesis [54]. Human homeostatic iron regulatory protein (HFE) and ATPase copper transporting beta (ATP7B) germline gene mutations can cause chronic liver injury and progression into HCC by excessive iron (hemochromatosis) and copper (Wilson disease) accumulation in the liver, respectively [53]. Germline mutations together with extrinsic risk factors can significantly increase the risk for HCC. In the case of NAFLD-HCC, germline telomerase reverse transcriptase (TERT) mutations may determine the progression of NAFLD-cirrhosis into HCC [55]. SNPs in the genes encoding TNF-α [56], AFP [57], Toll-like receptor (TLR) 2 [58], microRNA(miR)-146a and 196a-2 [59] and IL-1β [60] are known to promote the pathogenesis of HCC. Polymorphisms in the vascular endothelial growth factor (VEGF) gene can promote the recurrence of HCC even after liver transplants [61]. Clearly there is an important genetic component that predisposes individuals to the development of HCC [62].

### 2.2. Tumor Microenvironment and Molecular Pathogenesis of HCC

The hepatic tumor microenvironment (TME) is a complex mixture of tumor and stromal cells embedded in the extracellular matrix (ECM) [63]. A significant shift in the composition of the ECM occurs during the progression of liver injury to liver fibrosis and subsequently to HCC [64]. Hepatic stellate cell (HSC)-derived myofibroblasts are the primary source of fibrillar collagen and basement membrane proteins for the development of liver fibrosis [65]. Excessive secretion of ECM components during hepatic fibrosis generates a hypoxic environment by reducing the O_2_ permeability into the fibrotic tissue. To overcome hypoxia, angiogenesis is promoted by increased expression of hypoxia-inducible factor 1 (HIF-1) [66], VEGF, angiopoietin (ANGPT) 1, ANGPT2 and basic fibroblast growth factor (bFGF), creating an abnormal and dysfunctional vascular network in the tumor (Figure 1) [67]. HIF-1 also promotes HCC cell immortality, invasion and metastasis [66]. IL-6, IL-10, VEGF, TGF-β and macrophage colony-stimulating factor (M-CSF) in the TME disrupt the normal function of dendritic cells and may enable tumor immune evasion. Tumor-associated macrophages (TAM) in the TME secrete multiple cytokines that regulate dendritic cell (DC) functions. TAM also regulates tumor growth, invasion, angiogenesis and metastasis by the secretion of growth factors, cytokines, chemokines and enzymes [63]. Collectively, the results of these studies indicate the importance of the TME in survival and progression of HCC.

Somatic gene mutations and epigenetic changes in hepatocytes are the primary drivers of HCC pathogenesis (Figure 1). Mutations in telomerase enzyme components TERT or TERC [68,69] and tumor suppressor gene TP53 [70] diminish the integrity of the hepatic cellular genome during HCC. Activation of the wingless/int-1 (Wnt) signaling pathway by mutations in CTNNB1 [71], axis inhibition protein (AXIN) 1, AXIN2, adenomatous polyposis coil (APC) and zinc and ring finger 3 (ZNRF3) genes promotes the uncontrolled growth of hepatic cells [72]. TGF-β, tyrosine kinase receptors (TKRs) and JAK/STAT are the other main cellular signaling pathways distorted during HCC pathogenesis [73]. Chronic liver injury during HCC progression induces TGF-β expression that promotes HSC differentiation into a myofibroblast phenotype to promote fibrogenesis by ECM protein secretion [74]. TKRs regulate a wide range of cellular functions including, cell growth, motility, differentiation and metabolism [75]. Dysregulation of the JAK/STAT cascade in HCC can promote cell growth, cell regeneration, apoptosis evasion, angiogenesis and metastasis [76].

Apart from genetic point mutations, genomic instability in HCC can also result from chromatin structure modifications. Copy number variation (CNV) is a frequent chromatin structure modification reported in HCC. Wang el al. detected 29 recurrently amplified and 22 recurrently deleted chromosome regions in HCC tumors [77]. Amplified regions contained oncogenes, such as cyclin D1 (CCND1) and MET, while deleted regions frequently housed tumor suppressor gene, including cyclin-dependent kinase inhibitor (CDKN) 2A, and CDKN2B [77]. Chromatin remodeling is essential for gene transcription, DNA replication, and DNA repair [78] that is governed by multiple protein complexes. Mutations in chromatin regulator SMARCA4, AT-rich interaction domain (ARID) 1A, ARID1B, ARID2, myeloid/lymphoid leukemia (MLL), MLL3, and bromodomain PHD finger transcription factor (BPTF) genes contribute to HCC [79,80].

## 3. Association of Gut Microbiota with the Pathogenesis of HCC

**Figure 2:**
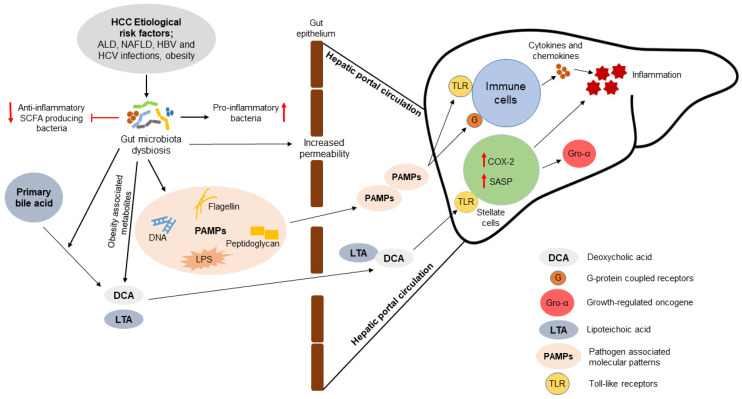
Association of gut microbiota with the pathogenesis of hepatocellular carcinoma (HCC). Chronic prevalence of the HCC etiological risk factors causes gut microbiota (GM) dysbiosis by depleting the beneficial anti-inflammatory short-chain fatty acid (SCFA) producing bacteria and promoting the growth of pro-inflammatory bacteria. GM dysbiosis disturbs the gut epithelial integrity and promotes the leakage of pathogen-associated molecular patterns (PAMPs) into the hepatic portal circulation. PAMPs reaching the liver promote inflammation by stimulating the immune cells to produce cytokines and chemokines through Toll-like receptors (TLR) and G-protein coupled receptors (G). The obesity-associated gut microbial metabolite lipoteichoic acid (LTA) and secondary bile acid deoxycholic acid (DCA) can increase the expression of cyclooxygenase-2 (COX-2) and senescence-associated secretory phenotype (SASP) in the hepatic stellate cells. Increased expression of COX-2 and SASP promote hepatic inflammation and upregulate the expression of growth-regulated oncogene-α (Gro-α). GM can convert primary bile acids to secondary bile acids including DCA. Up-pointed red arrows represent upregulated gene expression/increased pro-inflammatory bacteria population, down-pointed red arrow represents decreased anti-inflammatory SCFA producing bacteria population, and flathead red arrow represents the growth inhibition of anti-inflammatory SCFA producing bacteria. ALD, alcoholic liver disease; HBV, hepatitis B virus; HCV, hepatitis C virus; NAFLD, non-alcoholic fatty liver disease.

Since the liver is directly connected with the gut through portal circulation, the gut-liver axis can contribute to the pathogenesis of HCC by exposing the liver to PAMPs, such as bacterial lipopolysaccharides (LPS), DNA, peptidoglycans and flagellin (Figure 2). Many of the risk factors of HCC, including HBV and HCV infections, ALD and NAFLD, stimulate GM dysbiosis and increase intestinal permeability (Figure 2) [12,81]. The nature of HCC-associated GM dysbiosis is determined by HCC etiology. There is a considerable difference between the GM dysbiosis in patients with HBV-related HCC compared to non-HBV/non-HCV-related HCC (NBNC-HCC). The GM species richness of the HBV-HCC patients is significantly high compared to that of NBNC-HCC patients and healthy controls [82]. In NBNC-HCC, the GM shift favors the promotion of inflammation in the host. Pro-inflammatory bacteria (e.g., *Escherichia*, *Shigella* and *Enterococcus*) are increased in the GM while the anti-inflammatory SCFA-producing bacteria (e.g., *Faecalibacterium*, *Ruminococcus*, and *Ruminoclostridium*) are depleted in the NBNC-HCC patients [82]. Acetate, propionate and butyrate are the major SCFAs produced by GM. Intestinal epithelial cells, immune cells, and adipocytes express G-protein coupled receptors (GPCR) that are stimulated by SCFAs. Both propionate and butyrate can inhibit the proliferation of lymphocytes, thus opposing inflammation. This is achieved by the stimulation of GPCR41 receptors. SCFAs also inhibit histone deacetylase (HDAC) and cytokine expression by T-cells and activate Treg cells [83]. The nature of HCC-associated dysbiosis is also influenced by host physiological factors. Analysis of the fecal samples from HCC-cirrhotic patients revealed a significant depletion of GM richness in overweight patients compared to the patients with normal body weight [84]. Thus, the nature of GM dysbiosis in HCC is significantly dependent on the etiology and host physiological factors.

The host immune system is sensitive to the GM and GM-derived metabolic products through Toll-like receptors (TLR) of the immune cells and metabolic cells including hepatocytes and adipocytes (Figure 2). Increased intestinal permeability leads to leakage of gut microbes and microbial products into the systemic circulation where they affect organ function [85]. There is a strong correlation between liver cancer and the level of flagellin and LPS antibodies in the blood [86]. Mechanistically, this involves PAMPs stimulation of TLR-mediated immune responses by releasing cytokines and chemokines. GM-derived LPS also activates Kupffer cells in liver through TLR and the subsequent secretion of inflammatory cytokines [85]. Lipoteichoic acid (LTA) is an obesity-induced Gram-positive gut microbial component that can promote obesity-associated HCC pathogenesis in mice through TLR signaling. Together with deoxycholic acid (DCA), another obesity-induced gut microbial metabolite, LTA upregulates the expression of COX-2 and senescence-associated secretory phenotype (SASP) factors in HSC through TLR2 signaling [87]. Secretion of SASP factors can promote HCC by expression of inflammatory cytokines (IL-1β and IL-6) and growth-regulated oncogene (Gro)-α. Overexpression of COX-2 suppresses the antitumor immunity by stimulating prostaglandin E2 (PGE2) receptor 4 on immune cells. This suppression of antitumor immunity may occur by depletion of the CD103^+^ DC population and a lower CD8^+^ T-cell to Treg ratio. Overexpression of COX-2 and increased PGE2 production is common in HSC of non-cirrhotic NASH-HCC patients [87]. Furthermore, the potential of GM to convert primary bile acids (e.g., cholic acid and chenodeoxycholic acid) into secondary bile acids (e.g., DCA) may promote the progression of NASH to HCC. DCA can induce the carcinogenic mTOR signaling pathway in the HepG2 cell [88].

## 4. Probiotic Bacteria-Mediated Mechanisms to Attenuate HCC

The attenuation of HCC pathogenesis by probiotic bacteria has been described in vitro and in vivo (Table 1). Probiotics can favor the growth of GM bacteria that produce anti-inflammatory metabolites with tumor suppression activity. Prohep is a novel mixture of probiotic bacteria that promotes the growth of the *Prevotella* genus that are propionate producers and the *Oscillibacter* genus, which is associated with IL-10 producing Treg cell homeostasis. Compared to controls, mice fed Prohep and subcutaneously injected with Hepa1-6 murine hepatoma cells had a 40% reduction in tumor growth [89]. As well, the expression of inflammatory cytokine IL-17 in tumors was significantly reduced by the suppression of Th17 cell population and Th17 cell infiltration from the intestine and peripheral circulation. Moreover, probiotic supplementation upregulated the expression of the anti-inflammatory cytokines IL-10, IL-13 and IL-27. Downregulation of the angiogenic factors and receptors, VEGFA, Fms related receptor tyrosine kinase 1 (FLT-1), ANGPT2, and kinase insert domain receptor (KDR) was also detected in the mice fed the Prohep probiotic mixture [89].

**Figure 3:**
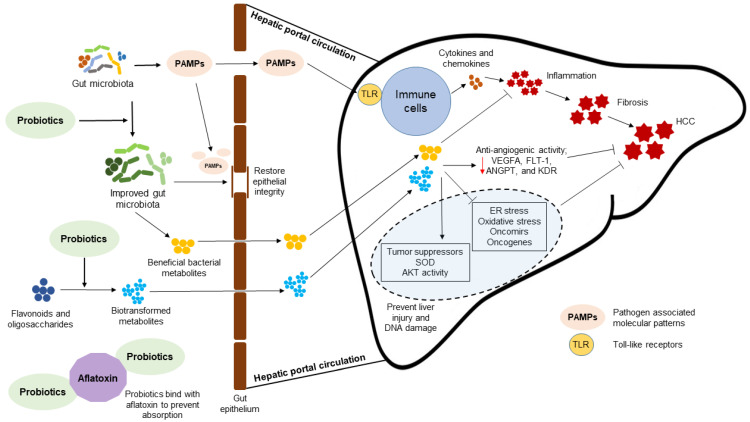
Mechanisms for reduction of hepatocellular carcinoma (HCC) by probiotic bacteria. Probiotic bacteria can enrich the gut microbiota (GM) and prevent the HCC-associated GM dysbiosis. Prevention of GM dysbiosis restores gut epithelial integrity and lowers the levels of pathogen-associated molecular patterns (PAMPs) in the circulation, and prevent hepatic inflammation caused by the stimulation of hepatic immune cells through PAMPs-induced Toll-like receptor (TLR) signaling. Probiotic bacteria can biotransform non-nutritional dietary components (e.g., flavonoids and oligosaccharides) into beneficial metabolites potent in HCC prevention. These metabolites prevent hepatic injury and DNA damage by promoting the expression of tumor suppressor genes protein kinase B (AKT) and superoxide dismutase (SOD), while preventing endoplasmic reticulum (ER) stress, hepatic oxidative stress and activation of oncogenes. Furthermore, these metabolites possess anti-inflammatory and anti-angiogenic properties. Probiotic bacteria can prevent aflatoxin-mediated HCC pathogenesis by binding with aflatoxin and preventing its absorption into body. Down-pointed red arrow represents downregulated gene expression and flathead arrows represent inhibitory effects. ANGPT, Angiopoietin; FLT-1, Fms related receptor tyrosine kinase 1; KDR, kinase insert domain receptor; VEGFA, vascular endothelial growth factor alpha.

Microbial PAMPs can promote HCC development through TLR-mediated inflammatory responses. Supplementation with probiotic bacteria attenuates HCC pathogenesis by downregulating the expression of TLR-induced inflammation in liver (Figure 3). Wistar rats with thioacetamide-induced liver cirrhosis had low expression of TLR4 and reduced liver damage when supplemented with *Lactobacillus* (*L.*) *plantarum* probiotic bacteria [93]. The expression of chemokine ligand 9 (CXCL9) and phosphatidylinositol 3,4,5-trisphosphate RAC exchanger 2 (PREX-2) was also low in rats supplemented with the probiotic bacteria. CXCL9 is known to promote the HCC invasion through PREX-2, therefore downregulation of the TLR4 and CXCL9/PREX-2 by probiotic bacteria could suppress the development of liver cirrhosis in the thioacetamide-treated rat model [93].

The ability of probiotic bacteria to promote the epigenetic modulation of host gene expression is beneficial to mitigate the pathogenesis of HCC [97]. The bacterial modulation of host gene expression is evident by the crosstalk between host and GM where gene expression is regulated by different mechanisms including DNA methylation and histone modification [105]. Probiotic bacteria *L. acidophilus* and *B. bifidum* can reduce the expression of oncomirs (miR-155 and miR-221) and the oncogenes BCL2-like 2 (Bcl-w) and Kristen rat sarcoma viral oncogene homolog (KRAS) in the liver of mice treated with the colon carcinogen azoxymethane. Moreover, the tumor suppressor miR-122 and tumor suppressor gene transcription factor PU.1 were overexpressed in mice supplemented with these probiotic bacteria [97]. Since acquired genetic mutations play a key role in HCC pathogenesis, probiotic supplementation may reduce the risk of HCC by protecting the hepatocyte genome. For instance, *L. paraplantarum* probiotic bacteria can reduce the diabetes-induced DNA damage in the livers of albino Wistar rats [94]. This reduction in DNA damage possibly resolves hepatic oxidative stress by restoring superoxide dismutase (SOD) activity in the diabetic rats. Moreover, probiotic supplementation of diabetic rat ameliorated hepatocyte injury by restoring Akt activity and preventing the degradation of pro-caspase 3. Interestingly, *L. paraplantarum* supplementation reduced liver inflammation and fibrogenesis by downregulating the CCAAT enhancer binding protein β (C/EBP/β) and α-2 macroglobulin (A2MG) expressions. C/EBP/β is a cytokine-inducible transcription factor expressed in hepatocyte differentiation and hepatic inflammation that facilitates fibrogenesis by upregulating the production of acute phase response reactants and connective tissue proteins. A2MG is a positive acute phase response protein that can promote fibrosis by inhibiting the catabolism of liver matrix proteins [94]. A novel probiotic mixture of *S. cerevisiae* and *L. acidophilus* enriched with selenium and glutathione synergistically prevented CCl_4_-induced liver fibrosis by the activation of silent information regulator 1 (SIRT1) in hepatocytes. SIRT1 is a member of class III group of HDAC. Activation of SIRT1 can ameliorate the hepatic oxidative stress, ER stress and inflammation induced by CCl_4_ in the rat livers as indicated by reduced serum ALT and AST activities [95].

The antiviral activity of probiotic bacteria may diminish HCC development by preventing chronic HBV infection. A cell extract of *B. adolescentis* restricted the growth of HBV in HepG2 cells and the secretion of HBV surface antigen (HBsAg) by 50% by inhibiting the expression of the HBsAg gene at the transcriptional level. Even though the intracellular level of HBV DNA was not significantly reduced by the probiotic treatment, the availability of extracellular HBV DNA was significantly reduced. The antiviral activity of *B. adolescentis* in HepG2 cells is governed by the activation of mycovirus resistance A (MxA) through upregulation of STAT1. MxA protein inhibits viral replication by binding to and degrading viral nucleocapsids and other viral components [90]. Treatment of HepG2 cells with extracellular (culture medium) extract of *L. bulgaricus* also reduced the viral load and cellular degeneration [91].

Supplementation with probiotic bacteria can also improve liver function during HCV infection. Heat-treated *E. faecalis* reduced the serum levels of liver damage markers ALT and AST in HCV-positive subjects. However, this probiotic bacteria failed to reduce the HCV viral load in the subjects [99]. Moreover, supplementation with probiotic bacteria *L. acidophilus* and *Bifidobacteria* spp. improved by 25% the response to HCV treatment by pegylated interferon (IFN)-α and ribavirin in the chronic HCV patients [100].

NAFLD is another major etiological risk factor of HCC pathogenesis. Supplementation with the probiotic bacteria *L. acidophilus* and *B. lactis* can ameliorate liver damage in NAFLD patients as indicated by reduced serum levels of ALT, AST, and total cholesterol [101]. In obese NAFLD patients, probiotic administration significantly reduced body weight and total body fat content. Moreover, probiotics reduced hepatic inflammation in obese NAFLD patients by downregulating the pro-inflammatory cytokine TNF-α [102]. Similar results were reported by Duseja et al. [103] for NAFLD patients following recommended life style changes (exercise and dietary). Multi-strain probiotic (*Lactobacillus* and *Bifidobacterium*) supplementation can significantly improve liver histology in these patients by reducing the hepatic damage caused by hepatocyte ballooning, hepatic fibrosis and lobular inflammation [103]. Hepatoprotective and anti-inflammatory effects of probiotic bacteria in NAFLD patients appeared to have resulted from changes in the GM. For example, a probiotic diet ameliorated diet-induced loss of GM richness, colonization resistance and gut epithelial barrier function in rats fed with a high-sucrose and high-fat (HSHF) diet. Restoration of GM and gut epithelial barrier function prevented NAFLD progression by reducing the serum LPS level and inhibiting the activation of TLR4-mediated hepatic inflammation [96].

Contamination of food with aflatoxin is an etiological risk factor of HCC in developing countries. A probiotic yogurt containing *S. thermophilus*, *L. rhamnosus*, and *W. cibaria* significantly reduced the presence of aflatoxin metabolites in the urine collected from children consuming maize contaminated with aflatoxins B1. This reduction of aflatoxin metabolites in urine may have resulted from the binding to probiotic bacteria leading to reduced intestinal absorption (Figure 3). *L. rhamnosus* and *W. cibaria* probiotic bacteria bind aflatoxin through the cell wall peptidoglycans [98].

The potential of probiotic bacteria to biotransform dietary components into metabolites with anticancer properties can be beneficial to prevent HCC (Figure 3) [106]. Probiotic bacteria *L. rhamnosus* can biotransform cranberry flavonoids into simple phenolic acids. Treatment of HepG2 cells with these biotransformed flavonoids inhibited cell proliferation by depletion of ATP. The concentration of biotransformed flavonoids required to inhibit HepG2 cell proliferation was significantly lower compared to that of non-biotransformed flavonoids [92]. Similarly, combined administration of a multi-strain probiotic mixture together with fructo-oligosaccharide improved the physiological parameters of non-obese NAFLD patients. This synbiotic approach significantly reduced hepatic steatosis and fibrosis in tested NAFLD patients. Moreover, supplementation with the synbiotic effectively reduced serum AST, fasting blood sugar, triacylglycerol and the inflammatory mediators, C-reactive protein (CRP), and NF-κB [104]. Scientific investigations reveal that potential exists to develop cancer-preventive synbiotic functional foods [107]. However, more evidence is required through randomized, double-blind, placebo-controlled trials to demonstrate the benefits of dietary supplementation of synbiotics to prevent and reverse pathogenesis of HCC development. Intensive studies are required to identify bioactive probiotic metabolites of specific dietary phytochemicals and to understand the possible mechanism(s) that might be involved in the interaction of these post-biotics with the host.

## 5. Summary

The pathogenesis of HCC is a complex process governed by multiple etiological risk factors; HBV and HCV infections, alcohol abuse, NAFLD/NASH, genetic mutations, and chronic aflatoxin ingestion. Chronic prevalence of a single or combination of these risk factors induces hepatic injury that could subsequently progress into fibrosis, cirrhosis, and eventually HCC. Probiotic bacteria can reduce the risk of HCC pathogenesis through multiple mechanisms such as modulation of the host GM and prevention of dysbiosis-associated endotoxemia, maintenance of gut epithelial barrier function, and inhibition of translocation of gut bacteria and PAMPs into the systemic circulation. Probiotics promote the growth of beneficial microbes producing anti-inflammatory metabolites that could relieve hepatic oxidative stress in HCC by increasing the expression of antioxidant enzymes. The antiviral activity of probiotics can be beneficial to mitigate HCC risk by preventing chronic HBV and HCV infections. Moreover, probiotics prevent hepatic lipotoxicity by ameliorating obesity. The anti-angiogenic properties of probiotic bacteria are associated with downregulation of VEGF and angiogenic factors VEGFA and ANGPTs. Interestingly, probiotics can upregulate the expression of tumor suppressors and inhibit the expression of oncogenes that contribute to HCC pathogenesis. Finally, probiotic bacteria can biotransform non-nutritional dietary components such as proanthocyanidin into simpler metabolites with anticancer effects against HCC. Designing synbiotics with enhanced anticancer properties may contribute to the development of dietary approaches and adjunct therapies as preventive measures for HCC.

## Figures and Tables

**Table 1:** Probiotics-mediated mechanisms for the reduction of hepatocellular carcinoma risk.

Probiotic Bacteria	Experimental Model	Findings	Reference
**In vitro studies**			
(1) *B. adolescentis* SPM0212	HepG2.2.15 cells were incubated with the probiotic cell extract and HBV for 24 h.	The probiotic cell extract inhibits the replication of HBV virus by activation of MxA protein through upregulation of STAT1.	[90]
(2) *L. bulgaricus* 761N	HepG2 cells were incubated with a extracellular extract of probiotic bacteria and HCV for 96 h.	Treatment with culture media extract of probiotic bacteria significantly reduced the HCV viral load and HepG2 cell death.	[91]
(3) Cranberry proanthocyanidin extract biotransformed by *L. rhamnosus*	HepG2 cells were incubated with biotransformed proanthocyanidins (10–500 µg/mL) up to 48 h.	Biotransformed proanthocyanidins inhibit the proliferation of HepG2 cells by depleting mitochondria. The effective concentration of biotransformed proanthocyanidins is significantly low compared to the non-biotransformed material.	[92]
**In vivo studies**			
(4) Prohep, a novel probiotic mixture of *L. rhamnosus*, *E. coli* Nissle 1917, and heat inactivated VSL#3 (1:1:1)	Male C57BL6/N mice (5–6 weeks) were fed with the probiotic mixture ad libitum and subcutaneously injected with murine hepatoma cells Hepa1-6.	Supplementation with the probiotic mixture modulated GM to suppress the tumor growth by downregulating inflammatory cytokine IL-17 and upregulating the expression of anti-inflammatory cytokines. Probiotic supplementation also downregulated the expression of angiogenic growth factors and receptors.	[89]
(5) *L. plantarum* EMCC-1039	Wistar rats were supplemented with 1.2 × 10^9^ cfu/mL of probiotic bacteria daily and liver cirrhosis was induced by administration of thioacetamide (200 mg/kg b.w. intraperitoneal) three times a week.	Probiotic supplementation attenuated thioacetamide-induced cirrhosis in rat livers by suppressing the expression of TLR4, CXCL9, and PREX-2.	[93]
(6) *L. paraplantarum* BGCG11	Albino Wistar rats were intraperitoneally injected with streptozotocin (40 mg/kg b.w./day) for 5 days and supplemented with probiotic bacteria (1 × 10^8^ cfu/day) for 4 weeks.	Probiotic supplementation reduced hepatic DNA damage by restoring the SOD activity in diabetic rats. Probiotics also reduced hepatic inflammation and liver fibrosis by restoring Akt signaling and preventing the degradation of pro-caspase 3.	[94]
(7) A novel probiotic mixture of *Saccharomyces cerevisiae* and *L. acidophilus* enriched with selenium and glutathione	Liver fibrosis in male Wistar rats was induced by intraperitoneal injection of CCl_4_ (2 mL/kg) twice a week for seven weeks. Rats were then supplemented with probiotic mixture (1 g/kg b.w./day) for seven weeks. (Daily intake of, selenium, 38.4 µg/kg b.w.; glutathione 34.1 mg/kg b.w.; *S, cerevisiae*, 1 × 10^10^ cfu; *L. acidophilus*, 1 × 10^10^ cfu)	Probiotic bacteria together with selenium and glutathione synergistically reduced liver damage and fibrosis. The probiotic mixture inhibited CCl_4_-induced oxidative stress, ER stress and inflammation by the activation of SIRT1.	[95]
(8) A probiotic mixture of *B. nfantis*, *L. acidophilus*, and *Bacillus cereus*	Male SPF SD rats on a HSHF diet were supplemented with the probiotic mixture for 12 weeks. (0.5 × 10^6^ cfu/day of *B. infantis* and *L. acidophilus*, and 0.5 × 10^5^ cfu/day *Bacillus cereus*)	Supplementation with probiotic bacteria ameliorated the loss of GM richness, colonization resistance and gut barrier function in rats fed with HSHF diet. This in turn reduced serum LPS levels and activation of TLR4-mediated immune response.	[96]
(9) *L. acidophilus* and *B. bifidum*	Male Balb/c mice (6 weeks) were supplemented with the two probiotic bacteria separately (1 × 10^9^ cfu/day) for five months. Ten days into the probiotic supplementation, mice were subcutaneously injected with the carcinogen azoxymethane (15 mg/kg b.w.) weekly for three weeks to induce colon cancer.	Probiotic supplementation downregulated the expression of oncomirs (miR-155 and miR-221) and oncogenes (Bcl-w and KRAS) in liver tissue. Moreover, probiotic supplementation upregulated the expression of tumor suppressor miR-122 and gene PU.1.	[97]
**Clinical studies**			
(10) A yoghurt with *S. thermophilus*, *L. rhamnosus* GR-1, and *Weissella cibaria* NN20	Children of 6–10 years old were provided 200 mL of the probiotic yogurt daily for 14 days.	Supplementation with the yogurt containing probiotic bacteria significantly reduced the urine availability of aflatoxin metabolites.	[98]
(11) Heat-treated *Enterococcus faecalis* FK-23	Long term supplementation (2700 mg/day up to 36 months) of HCV positive subjects with the heat-treated probiotic bacteria.	Heat-treated probiotic bacteria significantly reduced the serum levels of ALT and AST.	[99]
(12) A mixture of *L. acidophilus* and *Bifidobacteria* spp. probiotic bacteria.	Chronic HCV patients were fed the probiotic mixture (1 × 10^9^ cfu/day) daily for one month and subjected to pegylated IFN-α and ribavirin treatment weekly for 12 weeks.	Administration of probiotic bacteria increased the response rate to pegylated IFN-α and ribavirin treatment by 25%.	[100]
(13) A yogurt with *L. acidophilus* La5 and *B. lactis* Bb12	Adult NAFLD patients (23–63 years old) were fed 300 g of the probiotic yogurt for 8 weeks. (4.42 × 10^6^ cfu/g yogurt of *L. acidophilus* La5 and 3.85 × 10^6^ cfu/g yogurt of *B. lactis* Bb12)	Supplementation with probiotics ameliorated the NAFLD risk factors. Serum levels of ALT, AST, and total cholesterol is significantly reduced in the NAFLD patients supplemented with probiotic bacteria.	[101]
(14) A Probiotic mixture of *L. acidophilus* CBT LA1, *L. rhamnosus* CBT LR5, *L. paracasei* CBT LPC5, *Pediococcus. pentosaceus* CBT SL4, *B. lactis* CBT BL3, and *B. breve* CBT BR3	Obese NAFLD patients were supplemented with the probiotic mixture (1 × 10^9^ cfu/day) for 12 weeks.	Supplementation with the probiotic mixture significantly reduced the body weight, total body fat, total cholesterol and intra hepatic fat fraction of obese NAFLD patients. Probiotics administration also reduced the TNF-α expression in the NAFLD patients.	[102]
(15) A multi-strain probiotic mixture of *L. paracasei* DSM 24733, *L. plantarum* DSM 24730, *L. acidophilus* DSM 24735 and *L. delbrueckii subsp. bulgaricus* DSM 24734, *B. longum* DSM 24736, *B. infantis* DSM 24737, *B. breve* DSM 24732, and *S. thermophilus* DSM 24731	NAFLD patients complying with exercise and dietary recommendations were fed a multi-strain probiotic mixture (675 × 10^9^ cfu/day) for 12 months.	Probiotic supplementation improved the liver histology in NAFLD patients by reducing hepatocyte ballooning and hepatic fibrosis. Probiotic bacteria also reduced hepatic lobular inflammation and levels of ALT, adipocytokines, leptin and endotoxins.	[103]
(16) A synbiotic of *L. casei*, *L. rhamnosus*, *S. thermophilus*, *B. breve*, *L. acidophilus*, *B. longum*, and *L. bulgaricus* together with FOS	Non-obese NAFLD patients were supplemented with the synbiotic (0.4 × 10^9^ cfu of probiotics/day and 250 mg of FOS/day) daily for 28 weeks.	Supplementation with the synbiotic significantly reduced haptic steatosis and fibrosis in the non-obese NAFLD patients. Synbiotic administration also reduced the levels of liver damage marker and inflammatory mediators.	[104]

ALT, alanine aminotransferase; ANG2, angiopoietin 2; AST, aspartate aminotransferase; *B.*, *Bifidobacterium*; Bcl-w, BCL2 like 2; b.w., body weight; cfu, colony forming units; CXCL9, chemokine (C-X-C motif) ligand 9; FLT-1, Fms related receptor tyrosine kinase 1; GM, gut microbiota; HBV, hepatitis B virus; HCV, hepatitis C virus; hs-CRP, high-sensitive C-reactive protein; HSHF, high sucrose and high fat; IL, interleukin; KDR, kinase insert domain receptor; KRAS, Kristen rat sarcoma viral oncogene homolog; *L*., *Lactobacillus*; LPS, lipopolysaccharide; miR, microRNA; MxA, mycovirus resistance A; PREX-2, phosphatidylinositol 3, 4, 5 triphosphate RAC exchanger 2; PU.1, transcription factor PU.1; *S*., *Streptococcus*; STAT1, signal transducer and activator of transcription 1; SIRT1, silent information regulator 1; TLR4, Toll-like receptor 4; VEGFA, vascular endothelial growth factor A; IFN-α, interferon-α.
